# Blame does not keep patients safe

**Published:** 2019-09-10

**Authors:** Elmien Wolvaardt

**Affiliations:** 1 Editor: Community Eye Health Journal, International Centre for Eye Health, London School of Hygiene & Tropical Medicine, UK.

**A focus on systems and culture, rather than individuals, is ideal.**

Many organisations and health systems – even the legal system, to some extent – seek to blame individual health care workers if a patient suffers harm. The truth is that human error is inevitable: people make mistakes.^1^

The health care environment is highly complex. Many systems and processes, e.g., procurement, stock room management, record-keeping, staff rosters and clinical processes, and the people involved in them, must function effectively and in step with one another in order to ensure safe care and good outcomes.

Unless those involved in a medical error deliberately sought to cause harm, blaming and punishing them does not help. Instead, focus on the systems and processes that guide and support heath care workers. Are they adequate? Do they reduce the risk of errors? If not, how can they be improved? If the system doesn't change, the next person in the job will simply repeat the error. The World Health Organization Safe Surgery approach^2^ is an excellent example of minimising error by improving the system and framework.

## The ideal: a culture of safety and learning

Ideally, health care providers should establish a no blame culture, or ‘just culture’, in their organisation.

A just culture “considers wider systemic issues where things go wrong, enabling professionals and those operating the system to learn without fear of retribution.”^3^ This absence of retribution is crucial. If health workers sense that making an error will have a negative impact on their career, they will not report it. However, the absence of retribution may not be enough: health workers should be treated with respect and encouraged to speak up if something is wrong, as exemplified in the WHO Safe Surgery approach.^2^

For a health care culture to be ‘just’, it has to be one in which people “want to share what they know, because they can see how it will help to identify real, systemic causes of patient safety lapses.”^3^ Team members have to be confident that managers will work with them to deal with the underlying causes or contributing factors to an error,^4^ else they may lack the motivation to report errors and keep accurate records.

Creating a just culture requires leadership, joined-up thinking, collaboration with policy makers and professional bodies, good record-keeping, and human resources and administrative processes that reward quality, courage and honesty. A challenging, but worthwhile, task.

## Share your thoughts

If you have transformed your organisational culture, we want to hear from you. Write to **editor@cehjournal.org**.

## Useful resources

- Patient Safety Learning (**www.patientsafetylearning.org**) develop initiatives and resources (e.g. the Blueprint for Action^4^) to address systemic and cultural factors affecting patient safety and the handling of medical errors. Health workers can join in discussions and download or share resources with others, free of charge, at the Patient Safety Learning Hub: **www.pslhub.org**
- The Communication and Optimal Resolution (CANDOR) process is based on ‘just culture’ principles. The entire toolkit, including teaching resources, is available free of charge. **https://www.ahrq.gov/patient-safety/capacity/candor/modules.html**.
